# Small tumor necrosis factor receptor biologics inhibit the tumor necrosis factor-p38 signalling axis and inflammation

**DOI:** 10.1038/s41467-018-03640-y

**Published:** 2018-04-10

**Authors:** Violet R. Mukaro, Alex Quach, Michelle E. Gahan, Bernadette Boog, Zhi H. Huang, Xiuhui Gao, Carol Haddad, Suresh Mahalingam, Charles S. Hii, Antonio Ferrante

**Affiliations:** 1 Department of Immunopathology, SA Pathology, Women’s and Children’s Hospital, North Adelaide, SA 5006 Australia; 2 Discipline of Paediatrics, School of Medicine, Robinson Research Institute, University of Adelaide, Adelaide, SA 5001 Australia; 3 Faculty of Science and Technology, University of Canberra, Bruce, ACT 2601 Australia; 4 Discipline of Physiology, School of Medicine, University of Adelaide, Adelaide, SA 5001 Australia; 5 Emerging Viruses and Inflammation Research Group, Institute of Glycomics, Griffith University, Gold Coast, QLD 4222 Australia; 6 School of Biological Sciences, University of Adelaide, Adelaide, SA 5001 Australia; 7 School of Pharmacy and Medical Sciences, University of South Australia, Adelaide, SA 5001 Australia; 8 Present Address: Andrew Love Cancer Center, Geelong Hospital, School of Medicine, Deakin University, Geelong, VIC 3220 Australia; 9 Present Address: College of Medicine, University of Illinois, Chicago, IL 60612 USA

## Abstract

Despite anti-TNF therapy advancements for inflammatory diseases such as rheumatoid arthritis, the burden of diseases remains high. An 11-mer TNF peptide, TNF_70–80_, is known to stimulate selective functional responses compared to the parent TNF molecule. Here, we show that TNF_70–80_ binds to the TNF receptor, activating p38 MAP kinase through TNF receptor-associated factor 2. Using truncated TNFR mutants, we identify the sequence in TNFRI which enables p38 activation by TNF_70–80_. Peptides with this TNFRI sequence, such as TNFRI_206–211_ bind to TNF and inhibit TNF-induced p38 activation, respiratory burst, cytokine production and adhesion receptor expression but not F-Met-Leu-Phe-induced respiratory burst in neutrophils. TNFRI_206–211_ does not prevent TNF binding to TNFRI or TNF-induced stimulation of ERK, JNK and NF-κB. TNFRI_206–211_ inhibits bacterial lipopolysaccharide-induced peritonitis, carrageenan-induced and antigen-induced paw inflammation, and respiratory syncytial virus-induced lung inflammation in mice. Our findings suggest a way of targeting TNF-p38 pathway to treat chronic inflammatory disorders.

## Abstract (web summary)

Anti-TNF therapy has improved the treatment of inflammatory disease but can predispose to infection and malignancy. Here the authors show an anti-TNF biologic peptide that functionally and selectively targets the TNF-p38 pathway in multiple models of inflammation.

## Introduction

The pleotropic cytokine, tumour necrosis factor alpha (TNF), plays a major role in the pathogenesis of chronic inflammatory diseases. Consequently, the treatment of these diseases, in particular rheumatoid arthritis, Crohn’s disease and psoriatic arthritis, has been revolutionised by the availability of anti-TNF biologics such as monoclonal antibodies, e.g., infliximab, certolizumab, adalimumab and golimumab or soluble TNF receptors, e.g., etanercept^1^. However, the deleterious effects of neutralising the actions of TNF, including increased susceptibility to infection, remains a major setback for this type of therapy. Patients with rheumatoid arthritis receiving anti-TNF immunotherapy have the increased risk of fungal and bacterial infections, particularly of reactivating latent tuberculosis^1–4^, as well as non-melanoma skin cancers^5^. In paediatric and adult patients, combination therapy with anti-TNF biologics and other forms of immunosuppressives increased the risk of other types of malignancies^5,6^. Another approach in the treatment of chronic inflammation involves the targeting of the intracellular pathways used by the TNFR, in particular the p38 MAP kinase (MAPK). Unfortunately, this approach has encountered a number of major setbacks, including poor clinical responses and unexpected toxicity, resulting in suspension of clinical trials^7,8^. Thus, alternative approaches that target TNF and TNF-mediated signalling in chronic inflammatory conditions are needed.

It has previously been reported that short peptides of the TNF sequence promote different biological effects of the parent TNF molecule^9–11^. Of interest was that an 11-mer TNF peptide, TNF_70–80_, representing the amino acid sequence 70–80 of the TNF monomer, primed neutrophils and promoted immunity to Plasmodium, as well as *Mycobacterium bovis*, *Klebsiella pneumoniae* and *Aspergillus fumigatus* in vitro and/or in vivo^9–14^. Here, we show TNF_70–80_ binds to the TNFR and initiates signalling through TNF receptor-associated factor 2 (TRAF2) and p38 MAPK, leading to activation of the neutrophil respiratory burst. Using this information, we have generated peptides from the TNFRI sequence which interact with TNF_70–80_ and inhibit its ability to activate p38 and the respiratory burst. The TNFRI peptides block the inflammatory response in models of autoimmunity and infection. This provides a basis for developing cytokine receptor biologics with singular therapeutic potential.

## Results

### Activation of p38 MAP kinase by TNF_70–80_ biologic

**Fig. 1:**
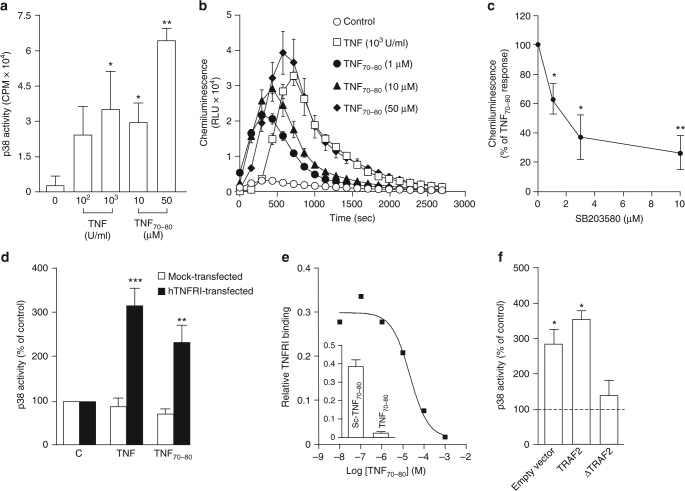
TNF_70–80_ activates p38 via the TNFRI. Neutrophils were treated with the indicated concentrations of either TNF or TNF_70–80_ and then incubated for 15 min before examining for p38 activation (**a**) or measuring chemiluminescence (**b**) over the 45-min period. The TNF_70–80_ induced chemiluminescence is inhibited by the p38 pharmacological inhibitor, SB203580. The neutrophils were pre-incubated with SB203580 for 10 min before the addition of TNF_70–80_. **c** TNF_70–80_ stimulates p38 activity in 70Z/3 pre-B cells, transfected with hTNFR1 (solid bars). Open bars represent mock-transfected cells. **d** Inhibition of biotin-labelled TNF_70–80_ binding to immobilised TNFR1 by unlabelled TNF_70–80_. **e** Peptide-TNFR1 binding was conducted as described under 'Methods'. Four-parameter curve fit yielded an IC_50_ value of 55 μM. (Inset) Lack of binding of scrambled peptide. The scrambled peptide and unlabelled TNF_70–80_ were both tested at 1 mM in the presence of 10 μM biotinylated TNF_70–80_. **f** TNF_70–80_ (10 μM) stimulated p38 activity in HEK 293T cells stably transfected with either wild-type TNF receptor-associated factor 2 (TRAF2) or an empty vector, but not in those transfected with a dominant-negative TRAF2 (∆TRAF2). Results are mean ± s.e.m. of four experiments. Significance of difference (two-tailed Mann–Whitney *U* test): *p < 0.05; **p < 0.01, ***p < 0.001

We have previously shown that peptides of different regions of the TNF sequence displayed selective biological properties in vitro and in vivo compared to the parent TNF molecule^9–11^. However, we have no understanding of how the peptide interacts with and activates cells. Here, we examined whether its stimulatory effects on the neutrophil respiratory burst is via the TNFR and p38 MAPK, both of which are required for a TNF-mediated response^15^. In these experiments, we used TNF and TNF_70–80_ at concentrations that we had previously found to enhance neutrophil responses^9–14^. The data demonstrate that in association with the activation of p38 MAPK, TNF_70–80_ stimulated the production of superoxide in a similar manner to TNF, measured as lucigenin-dependent chemiluminescence production (Fig. 1a, b). The TNF_70–80_-induced respiratory burst response was inhibited by the p38 inhibitor, SB203580 (Fig. 1c).

To understand how TNF_70–80_ caused p38 activation, we examined its interactions with TNF receptors. Since previous studies have found that TNFRI is coupled to p38 and is required for the effects of TNF on neutrophils^16,17^, we focused on TNFRI in the following investigations. To investigate whether the peptide acted via cell surface-expressed TNFR, we used the 70Z/3 pre-B cell line which lacks TNFRs. It has previously been demonstrated that 70Z/3 cells either do not bind TNF or lymphotoxin, or respond to TNF unless they have been transfected with TNFRI^18,19^. 70Z/3 pre-B cells were incubated with either TNF or TNF_70–80_ and the activation of p38 investigated. In these cells, both TNF and TNF_70–80_ failed to stimulate the activity of p38 (Fig. 1d). However, when the cells were transfected with vectors carrying TNFRI, both ligands caused the activation of p38 (Fig. 1d). These data demonstrate that TNF_70–80_ bound to and acted specifically via the TNFR. Evidence was also generated which showed that labelled TNF_70–80_ bound to soluble TNFRI could be competitively displaced with unlabelled TNF_70–80_ (Fig. 1e). To obtain further evidence that TNF_70–80_ enhanced the activity of p38 via a TNFR-mediated proximal signalling mechanism, we examined responses in HEK 293T cells which had been stably transfected with an empty vector, wild-type TRAF2 or a dominant-negative TRAF2 (∆TRAF2). The data show that TNF_70–80_ enhanced the activity of p38 in cells transfected with either wild-type TRAF2 or an empty vector, but not in those transfected with ∆TRAF2 (Fig. 1f). This shows that the activation of p38 by the peptide requires TRAF2.

### Development of TNFRI biologics with anti-TNF activity

**Fig. 2:**
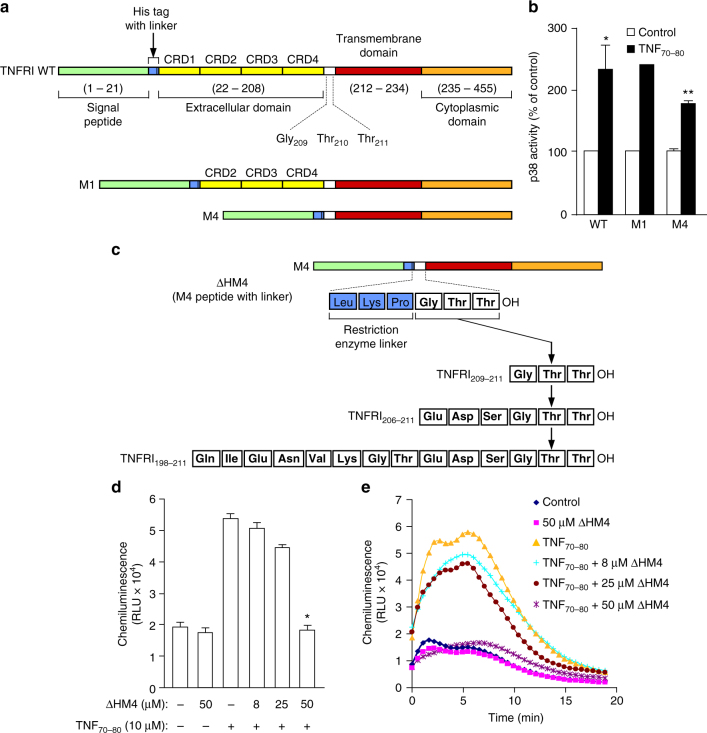
Development of TNFRI peptides with anti-TNF activity. **a** Schematic representation of TNFRI and mutants. WT wild type, CRD cysteine-rich domain, His histidine, linker restriction enzyme linker, M mutant. **b** p38 activation in cells transfected with WT or mutant TNFRI. 70Z/3 pro B cells transfected with WT TNFRI or truncated TNFRI mutants, M1 or M4, were treated with either HBSS or TNF_70–80_ (10 μM) for 5 min and p38 activity assayed. Results are mean ± s.e.m. of three separate experiments. Significance of difference between control and TNF_70–80_ (one-tailed one sample *t*-test): *p < 0.05, **p < 0.01. There was no difference between WT and M4 (*p* > 0.05, *n* = 3 experiments, two-tailed Mann–Whitney test). **c** Generation and schematic representation of TNFRI-derived peptides. The boldfaced text represents residues in the natural TNFRI sequence. Leu-Lys-Pro was introduced to generate a restriction site for coupling to the His-tag. **d** ∆HM4 (Leu Lys Pro Gly Thr Thr) inhibited the TNF_70–80_-induced CL production in neutrophils. Results are mean ± s.e.m. of four separate experiments. Significance of difference between TNF_70–80_ and TNF_70–80_ + ∆HM4 (Kruskal–Wallis test, followed by Dunn’s multiple comparison test): *p < 0.05. **e** Kinetics of TNF_70–80_-induced CL production in the presence of ∆HM4 peptide

Our data raise the tantalising possibility of a new approach to target the TNFR-p38 signalling pathway for therapeutic purposes if we are able to identify the region of TNFRI to which TNF_70–80_ binds. To achieve this objective, His-tagged truncation mutants of TNFRI were constructed by deleting the first (M1) or all four (M4) cysteine-rich domains (CRD) on the extracellular portion of TNFRI (Fig. 2a). Then, 70Z/3 pre-B cells lacking TNFR^18,19^ were transiently transfected with wild-type (WT) or a mutant of TNFRI. After 24 h, the cells were treated with either diluent or TNF_70–80_ (10 μM) and p38 activity assayed. The data showed that TNF_70–80_ caused ~2.5-fold increase in p38 activity in WT TNFRI- and M1 TNFR1-transfected cells and this effect was retained, albeit appearing reduced (*p* = 0.11, *n* = 3, two-sided *t* test), in cells transfected with the M4 TNFRI mutant (Fig. 2b).

Since the M4 mutant contains three remnant amino acid residues, GTT (TNFRI_209–211_), of the extracellular portion of TNFRI (Fig. 2c), our data imply that these residues represent the minimum region in TNFRI to which TNF_70–80_ binds and is required by TNF_70–80_ for activating p38. This therefore raised the possibility that peptides of this region might also block the function of TNF_70–80_. Thus, peptides corresponding to TNFRI_209–211_ were made and examined for ability to inhibit TNF_70–80_-induced respiratory burst in neutrophils using a lucigenin-based chemiluminescence (CL) assay. The first peptide, ∆HM4 (LKPGTT) (Fig. 2), composed of a three amino acid restriction enzyme linker sequence, LKP, that was introduced to couple the hexahistidine tag to the truncated TNFRI mutants and TNFRI_209–211_ (GTT) was synthesised and shown to cause a dose-dependent suppression in the ability of TNF_70–80_ to stimulate CL production in human neutrophils (Fig. 2d, e).

**Fig. 3:**
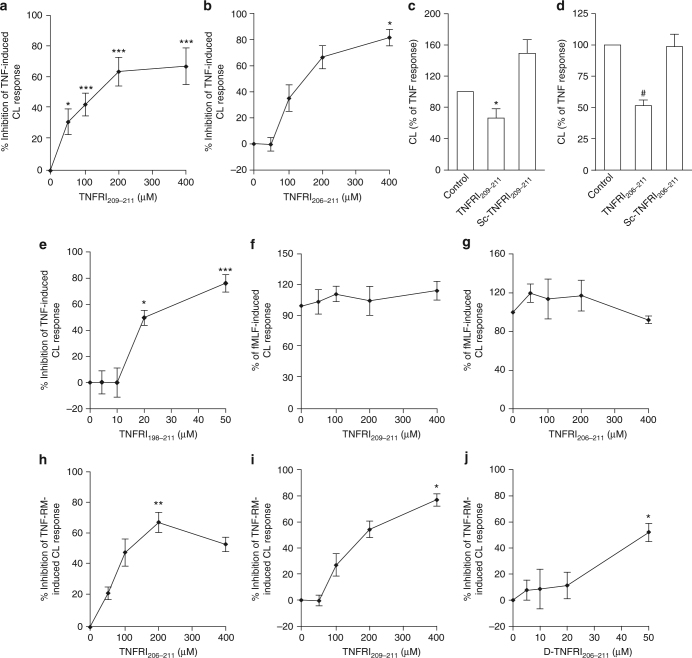
Inhibition of TNF-induced responses in neutrophils by TNFRI peptides. **a**, **b** Inhibition of superoxide production in neutrophils treated with TNF (1 ng/ml), which had been pre-treated with various concentrations of TNFRI_209–211_ or TNFRI_206–211_ for 30 min. **c**,** d** Lack of effect of scrambled peptides. **e** Inhibition of TNF-induced CL by TNFRI_198–211_. **f**, **g** Lack of effect of TNFRI_209–211_ and TNFRI_206–211_ on the fMLF-induced superoxide generation in neutrophils. The results are expressed as a % of fMLF-induced CL production. **h**, **i** Effect of TNFRI_206–211_ or TNFRI_209–211_ on TNF-rich medium (RM)-induced superoxide (chemiluminescence) production in neutrophils. TNF-RM (1:10 dilution in HBSS) was treated with TNFRI peptides and then tested for ability to stimulate neutrophil superoxide (chemiluminescence) production. Data represent the % inhibition of the TNF-RM-induced response. **j** The experiments in **h** were repeated with the D-form of TNFRI_206–211_. Data are presented as mean ± s.e.m. of the below-indicated number of experiments. Significance of difference between the presence and absence of peptide (Kruskal–Wallis test followed by Dunn’s multiple comparison test (*n* = 6 for **a**, *n* = 3 for **b**, *n* = 4 for **c**, *n* = 3 for **d** and **f**–**i** and *n* = 7 for **e** and **j**): *p < 0.05; **p < 0.01; ***p < 0.001; ^#^*p* = 0.055

Having obtained the above results with ∆HM4, it was important to exclude an effect due to the three linker amino acids. We therefore synthesised and tested the effect of TNFRI_209–211_ (GTT) on TNF-induced neutrophil CL production. Treating TNF with TNFRI_209–211_ compared to a control peptide (TGT) caused a dose-dependent inhibition of TNF-induced CL and an IC_50_ of 123 μM (Fig. 3a, c). A longer peptide, TNFRI_206–211_ (EDSGTT), that contained the three residues N-terminal to TNFRI_209–211_ but not the corresponding scrambled peptide, GEDTST, was effective at inhibiting TNF-induced CL production with an IC_50_ of 171 μM (Fig. 3b, d). These data demonstrate that the minimal TNFRI peptides, revealed by the above TNF_70–80_–p38 activity studies, were able to inhibit this function of TNF. In addition, we found that extending the sequence to include residues 198–211 of TNFRI (TNFRI_198–211_) (QIENVKGTEDSGTT) increased the anti-TNF action (IC50 of 50 μM) (Fig. 3e). The specificity of these receptor peptides was further supported by the finding that neither TNFRI_209–211_ (Fig. 3f) nor TNFRI_206–211_ (Fig. 3g) inhibited fMLF-stimulated CL production. Previously, we have demonstrated that neutrophil priming and activation of the respiratory burst by conditioned medium from *Staphylococcus aureus*-stimulated mononuclear leukocytes is due to TNF^20^. Treatment of the TNF-rich medium with either TNFRI_206–211_ or TNFRI_209–211_ also inhibited the neutrophil response with IC_50_ of 216 μM and 146 μM, respectively (Fig. 3h, i). A D-amino form of TNFRI_206–211_ was also an effective inhibitor of the TNF-induced CL response with an IC_50_ of 51 μM (Fig. 3j). Incubation with the peptides alone neither increased nor decreased the baseline CL in all experiments.

**Fig. 4:**
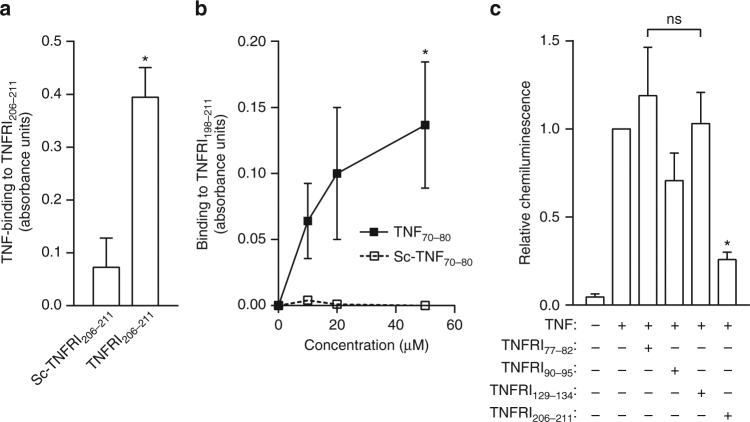
TNFRI peptides bind to TNF and TNF_70–80,_ and effects on respiratory burst. Examination of the ability of TNFRI peptides to bind to TNF and TNF_70–80_ was conducted in a solid phase assay as described in 'Methods'. **a** Binding to TNF and **b** binding to TNF_70–80_. **c** Shows the effectiveness of TNFRI_206–211_ compared to peptides derived from CRD1–CRD3 that do not contain the residues found in M4 at inhibiting the TNF-induced chemiluminescence response in human neutrophils. The peptides were each tested at 400 μM. Results are mean ± s.e.m. of three separate experiments. Significance of difference: *p < 0.05 or **p < 0.01 **a** between TNFRI_206–211_ and scrambled peptide (two-tailed Student’s *t* test), **b** between TNF_70–80_ and control (scrambled) peptide (Kruskal–Wallis test followed by Dunn’s multiple comparison test) **c** between TNF alone and TNF+ TNFRI peptide (one-way ANOVA followed by Dunnett’s multiple comparison test)

The receptor peptides showed binding to both TNF_70–80_ and TNF (Fig. 4a, b). To gain further evidence for the specificity of the above-described membrane proximal TNFRI peptides as anti-TNF agents, we also investigated whether peptides derived from the CRD of TNFRI, CDR 1, 2 and 3, were able to block the TNF-induced respiratory burst. The data show that these peptides, in contrast to the peptides in Fig. 3, did not affect the action of TNF (Fig. 4c), thus demonstrating their specificity.

### Inhibition of the inflammatory response

**Fig. 5:**
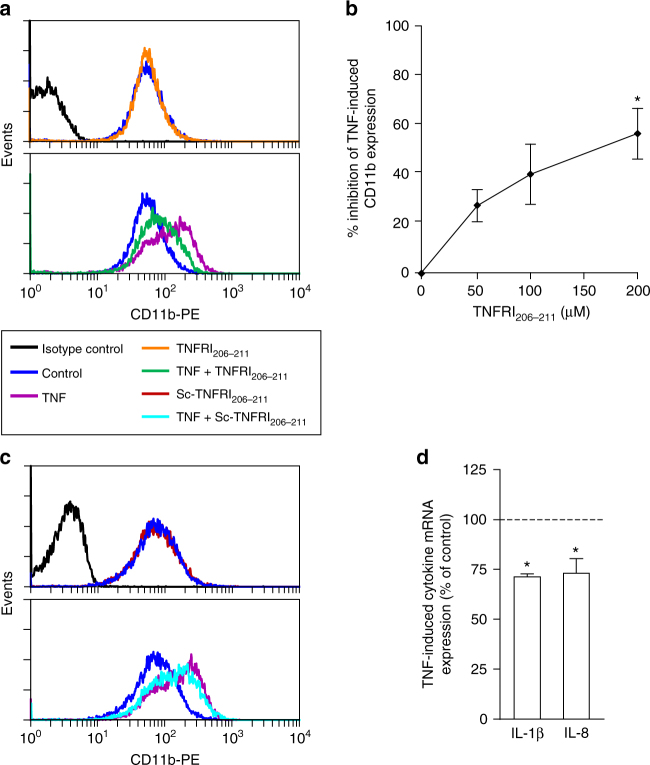
TNFRI peptides inhibit TNF-induced expression of CD11b and cytokines. In these studies, TNF (5 ng/ml) was pre-treated for 20 min with 200 μM TNFRI_206–211_ unless otherwise specified. **a**, **b** Inhibition of CD11b expression in neutrophils by TNFRI_206–211_. A representative histogram (**a**) representative of *n* = 3 experiments, and the concentration-dependent inhibition (**b**) are shown. Results are mean ± s.e.m. of four experiments. Significance of difference between TNF and TNF+ TNFRI peptide (Kruskal–Wallis test followed by Dunn’s multiple comparison test): *p < 0.01. **c** Lack of effect of scrambled TNFRI peptide on TNF-induced upregulation of CD11b expression. **d** Inhibition of TNF-induced IL-8/IL-1β mRNA expression in neutrophils by TNFRI_206–211_ Results are mean ± s.e.m. of *n* = 3 experiments. Significance of difference (one-tailed one sample *t*-test): *p < 0.05; compared to TNF alone

Apart from inhibiting the TNF-induced neutrophil oxygen radical production, the TNFRI peptides inhibited TNF-induced complement receptor type 3 (CD11b/CD18) upregulation in neutrophils (Fig. 5a–c) and TNF-induced neutrophil IL-1β and IL-8 production (Fig. 5d). These effects are indicative of the receptor peptide being an anti-inflammatory agent. We thus sought to examine its effects in in vivo inflammatory responses in mice.

**Fig. 6:**
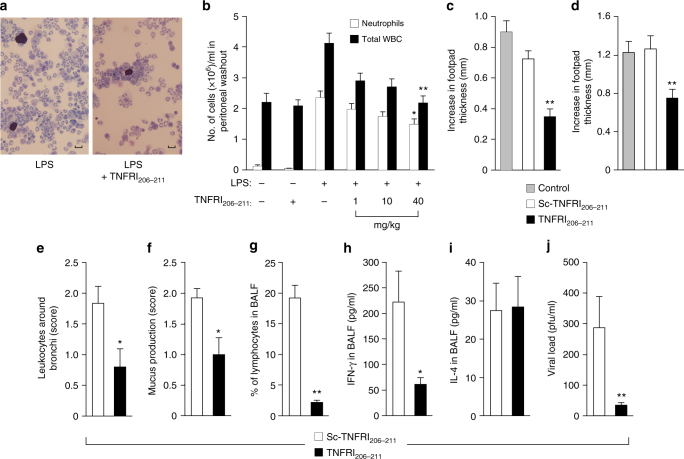
TNFRI_206–211_ inhibits inflammation. **a**, **b** Mice were injected ip with 50 µg LPS and after 2 h with TNFRI_206–211_. After 24 h peritoneal exudates were examined and a photomicrograph is presented in **a**, scale bar = 10 μm. Total white blood cells (closed bars) and neutrophils (open bars) were quantified (**b**). Results are mean ± s.e.m. of the indicated number of animals. For **b**, significance of difference between the presence and absence of peptide (Kruskal–Wallis test followed by Dunn’s multiple comparison test, *n* = 4 animals for HBSS control and *n* = 3 animals for TNFRI_206–211_ control group; *n* = 4 animals for LPS control, *n* = 7 animals for 1 mg/kg, *n* = 6 animals for 10 mg/kg and *n* = 5 animals for 40 mg/kg of TNFRI_206–211_ in the LPS-treated group): *p < 0.05, **p < 0.01. **c** Mice were injected with TNFRI_206–211_/scrambled peptide (10 mg/kg) or HBSS and SRBC solution, and 6 days later re-challenged with SRBC mixed with TNFRI_206–211_/scrambled peptide (12.5 mg/kg) or HBSS, or **d** with a mixture of carrageenan and TNFRI_206–211_/scrambled peptide (10 mg/kg) or HBSS into the hind footpad. Footpad thickness was measured at 24 h after the last treatment. Results are mean ± s.e.m. of the number of animals shown after the description of the statistical tests. Significance of difference between HBSS and TNFRI_206–211_ (**c** one-way ANOVA followed by Dunnett’s multiple comparison test, *n* = 9 animals for HBSS and scrambled peptide and *n* = 8 animals for TNFRI_206–211_; **d** Kruskal–Wallis tests followed by Dunn’s multiple comparison test, *n* = 5 animals for HBSS and TNFRI_206–211_, and *n* = 4 animals for scrambled peptide): **p < 0.01. **e**–**j** Mice were treated i.n. with 100 mg/kg of either control peptide (6 mice) or TNFRI_206–211_ (5 mice) on days −1, 1 and 3, and inoculated i.n. with RSV on day 0 and killed on day 5. H&E-stained lung sections were used to quantitate the number of leukocytes around the bronchi (**e**) and mucus production (**f**), and lymphocyte numbers (**g**) from BAL fluid (BALF), Giemsa-stained cytospins. IFNγ (**h**) and IL-4 (**i**) were measured by ELISA. **j** Lung viral load was determined by immunostaining. Results are presented as mean ± s.e.m. Significance of difference (Mann–Whitney test): *p < 0.05; **p < 0.01

The inflammatory reaction, number of infiltrating leukocytes and neutrophils, induced by the intraperitoneal (i.p.) injection of bacterial LPS was inhibited by TNFRI_206–211_ (Fig. 6a, b). Similar inhibition was seen in a chronic inflammation model induced by sheep red blood cell antigens (Fig. 6c) and an acute inflammation model of carrageenan-induced paw inflammation  in mice (Fig. 6d).

The effectiveness of TNFRI_206–211_ as an anti-inflammatory agent was further demonstrated in an acute respiratory syncytial virus (RSV)-induced inflammatory mouse model^21,22^. Mice were treated intranasally (i.n.) with TNFRI_206–211_ or the respective scrambled peptide, 1 day prior to an intranasal challenge with 1 × 10^6^ plaque forming units of RSV, and then 1 and 3 days post viral challenge. Mice treated with TNFRI_206–211_ had significantly reduced numbers of leukocytes around the bronchi (Fig. 6e) and mucus production in the lungs (Fig. 6f) compared with mice treated with the control peptide. There was also a reduction in lung epithelial thickness/inflammation score (~50%) (scrambled peptide: 1.83 ± 0.21; TNFRI_206–211_: 1.0 ± 0.27, mean ± s.e.m. of five mice/group, *p* > 0.05, Mann–Whitney *U* test) and in the number of leukocytes around the lung blood vessels (~70%) (scrambled peptide: 1.8+3 ± 0.40; TNFRI_206–211_: 0.7 ± 0.2, mean ± s.e.m. of five mice/group, *p* > 0.05, Mann–Whitney *U* test) although this did not reach significance. The anti-inflammatory effects of TNFRI_206–211_ were further supported by the findings that there was a significant reduction in lymphocyte numbers (Fig. 6g) and IFN-γ content (Fig. 6h) in the bronchoalveolar lavage (BAL) fluid. The TNFR peptide had no effect on IL-4 production (Fig. 6i), suggesting an effect through the inhibition of the Th1 responses. Neutrophil numbers in the BAL fluid were also reduced by TNFRI_206–211_. Neutrophils were detected in BAL fluids of five of six mice treated with the control peptide compared with only one of six mice treated with TNFRI_206–211_ (*p* < 0.05; one tail Fischer exact test). Interestingly, the anti-inflammatory effects of TNFRI_206–211_ were associated with a significant decrease in viral load in the lung (Fig. 6j), as in most cases impaired resistance to microbial pathogens has been reported in animals and humans treated with anti-inflammatory agents.

### TNFRI_206–211_ selectively inhibits the TNF-p38 axis

**Fig. 7:**
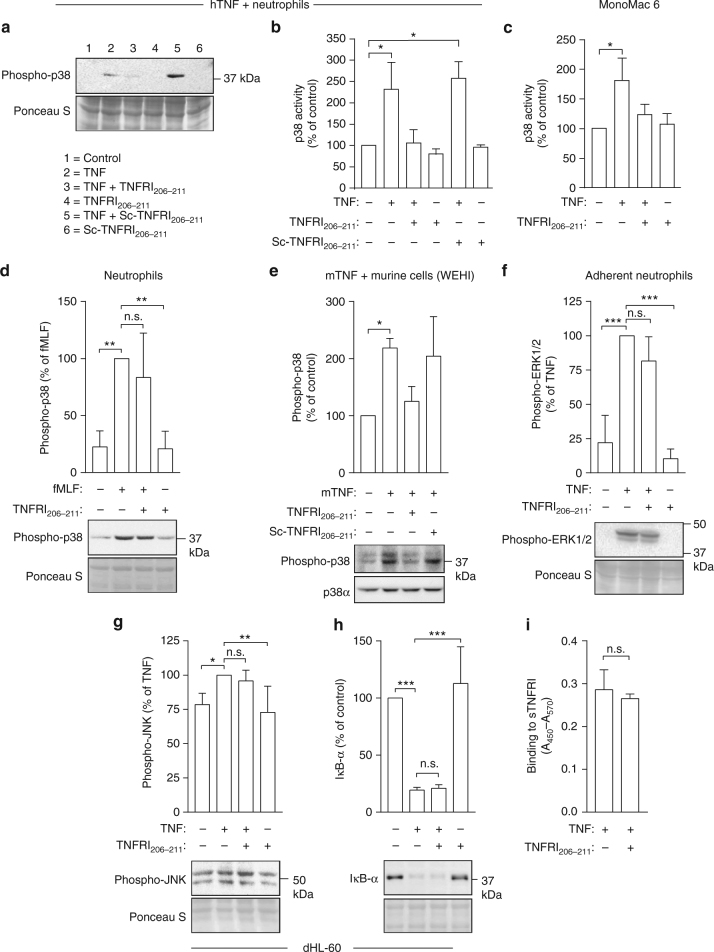
TNFRI_206–211_ selectively inhibits TNF-induced activation of p38. In these studies. TNF or FMLF was incubated with TNFRI_206–211_, then added to cells and examined for activation of MAP kinases and NF-κB (details are given in 'Methods'). **a** A representative western blot of three experiments shows that TNFRI_206–211_ but not the scrambled (Sc) TNFR_206–211_ inhibits TNF-induced p38 activation in human neutrophils and **b**, **c** show the quantitative data (mean ± s.e.m. of three experiments) from western blots of the inhibition of TNF-induced p38 activation in neutrophils and MonoMac 6 cells by TNFRI_206–211_. **d** The effects of TNFRI_206–211_ on FMLF-induced p38 activation in neutrophils (*n* = 3 experiments). **e** TNFRI_206–211_ inhibits p38 activation in WEHI cells in response to mouse TNF, which had been pre-treated with TNFR_206–211_. Results are presented as mean ± s.e.m. of three experiments. TNFR_206–211_ does not inhibit TNF-induced ERK1/ERK2 activation (**f**) (*n* = 3 experiments), JNK (**g**) (*n* = 5 experiments) and IκB-α degradation (**h**) (*n* = 3 experiments). **i** TNFR_206–211_ does not inhibit TNF binding to its receptor (TNFR1) (*n* = 4 experiments). Quantitated data are mean ± s.e.m. Significance of difference was analysed by one-way ANOVA with either Kruskal–Wallis test followed by Dunn’s multiple comparison test (**b**, **c**) or Brown–Forsythe testing followed by Dunnett’s multiple comparison test (**d**, **e**, **f**, **g**, **h**) or unpaired Student’s *t* test (**i**). *p < 0.05, **p < 0.01 and ***p < 0.001. n.s. not significant

The TNFRI peptides could exert their effects by blocking selectively the ability of TNF to signal via p38. To investigate this possibility, we pre-incubated TNF with TNFRI_206–211_ or scrambled peptide before adding these to neutrophils or mononuclear phagocytes (MonoMac 6 cells). The data in Fig. 7a, b demonstrate that TNFRI_206–211_ but not the scrambled peptide prevented TNF from inducing the activation of p38 in human neutrophils. This effect was reproduced in the MonoMac 6 cells (Fig. 7c). It is likely that TNFR_206–211_ has selectivity for this pathway because treatment of another neutrophil agonist, f-Met-Leu-Phe (FMLF) with TNFR_206–211_ did not inhibit the ability to activate p38 (Fig. 7d). To relate this to the effects of the receptor peptide on the inflammatory response, we examined its effect on mouse TNF acting on mouse WEHI-164 cells. The results demonstrated that TNFRI_206–211_ inhibits the activation of p38 in the murine system (Fig. 7e). These data imply that the peptides exerted their anti-inflammatory effects by blocking TNF signalling via the p38 pathway. Similar treatment of adherent human neutrophils or HL-60-derived neutrophils with TNF which had been pre-treated with the receptor peptide did not inhibit the activation of either the MAP kinases, ERK1/ERK2 (Fig. 7f) and JNK (Fig. 7g), or of NF-κB, the latter measured by examining the degradation of IκB-α (Fig. 7h). To investigate whether the TNF bound to TNFR_206–211_ was still able to bind to its receptor, we examined whether the receptor peptide prevented TNF from binding to the recombinant TNFRI. TNF bound similarly to TNFRI in the presence and absence of the receptor peptide (Fig. 7i).

## Discussion

Data from our previous reports demonstrated the unique biological characteristics of the short TNF mimetic peptide, TNF_70–80_, in vitro and in vivo^10,11^. Here, we show that this peptide stimulates the chemiluminescence response in human neutrophils via the activation of the MAP kinase, p38. Thus, the TNF-derived peptide stimulated the activation of p38 in the neutrophils and the chemiluminescence production was inhibited by the pharmacological p38 inhibitor, SB203580. Activation of p38 by TNF_70–80_ occurred via the TNF receptor, since cells lacking the TNFRI failed to show p38 activation, and TRAF2 was required for its ability to activate p38. Because these effects on cellular function are considered to be important elements in the generation of an inflammatory response, we used this peptide sequence to identify a complimentary region on the TNFR, which could be used to block the parent TNF molecule from stimulating these responses.

Using TNFR1-truncated constructs, we were able to identify a minimal region in TNFRI which supported the activation of p38 by TNF_70–80_. Interestingly, peptides of this minimal TNFRI sequence, TNFRI_209–211_ and TNFRI_206–211_, exhibited anti-TNF activity in vitro, as they inhibited the ability of TNF_70–80_ or TNF to induce a chemiluminescence response in neutrophils. Peptides representing other regions of the TNFRI such as those in the CRD did not inhibit this response. Furthermore, we showed that the TNFRI biologics could bind to both TNF_70–80_ and TNF. Further support for these interactions was obtained by the finding that the TNFRI biologics, TNFR_209–211_ and TNFRI_206–211_, inhibited TNF-mediated activation of p38 in both human and murine cells. These data demonstrate the efficacy and applicability of these TNFRI biologics to treat inflammation induced by a variety of agents.

In vivo, the TNFRI peptides inhibited LPS-induced peritonitis, carrageenan and antigen-induced inflammation and respiratory syncytial virus (RSV)-induced lung inflammation. The latter is particularly useful in gauging the actions of the TNFRI peptides, as both the effects on pathogenesis and susceptibility to the virus could be monitored. Importantly, treatment with the TNFRI peptides significantly reduced lung viral load, in addition to the suppression of virus-induced inflammation. This is the first demonstration that this new form of anti-TNF therapy has the potential to be used in the treatment of infection-induced pathogenesis as well as non-infectious inflammatory conditions.

The ability of TNFRI_206–211_ to bind TNF suggests a possible mechanism by which the TNFRI peptides exert their anti-TNF effects. Such an action would prevent TNF from fully engaging TNFRI, particularly around the region whose sequence is found in the TNFRI peptides. Consistent with this idea, it was necessary to preincubate TNF with a TNFRI peptide to enable the binding of the peptide to TNF prior to addition to cells. The data also show that whereas TNFRI_206–211_ bound TNF, the scrambled version that was devoid of inhibitory actions, did not bind. Furthermore, hexamers of TNFRI from CRD1–CRD3, unlike TNFRI_206–211_, did not prevent TNF from stimulating neutrophils to produce superoxide. This also demonstrates the specificity of residues 198–211 in TNFRI in coupling TNF-p38 to the neutrophil respiratory burst function.

Anti-TNF biologics have revolutionised the treatment of chronic inflammatory conditions such as rheumatoid arthritis^1^. However, this form of therapy is associated with increased susceptibility to infection and cancer^1–6^. Furthermore, the FDA has issued warnings against such use in paediatric patients between the ages of 4–17 owing to increased risk of malignancy^5,6^. Another approach in the treatment of chronic inflammation has involved the targeting of the intracellular pathways used by the TNFR. Although p38 inhibitors represent one of the most heralded form of therapies for the treatment of inflammation in recent times, clinical trials with a range of unrelated pharmacological p38 inhibitors for the treatment of rheumatoid arthritis and other chronic inflammatory conditions have shown poor clinical outcomes with transient immunosuppressive effects, associated with increased risk of severe infections, liver/brain/skin toxicity and other adverse effects^8,23,24^. These disappointing clinical data signal that systemic inhibition of p38 is an inappropriate form of treatment.

The p38 pathway plays a dual role in regulating the inflammatory response. Thus, blockade of the p38 pathway in murine macrophages or human synovial cells not only caused a decrease in the production of proinflammatory mediators such as MMP-13, KC, TNF and IL-6, but also the anti-inflammatory IL-10^25,26^. In addition, such blockade also resulted in a decrease in the expression of the MAP kinase phosphatase, DUSP-1^25,26^ that inactivates active p38 and active JNK. Consequently, p38 activation also initiates a negative feedback mechanism that limits the strength and duration of the function of this signalling pathway. Previous studies have demonstrated that the p38-mediated regulation of IL-10 and DUSP-1 expression is mediated by the downstream mitogen- and stress-activated kinases (MSK)1 and MSK2, two of the kinases that phosphorylate the transcription factors, CREB and ATF-1, that bind to the promoters of IL-10 and DUSP-1 and enhance their transcriptional activity^27^. Consistent with its role in mediating an anti-inflammatory action, p38 inhibition in vivo was found to cause disease exacerbation and prolonged the duration of disease in animal models of inflammatory diseases^25,26^. This is likely to be a key reason for the failure of drugs that target p38 in clinical trials.

The TNFRI peptides do not target p38 per se, unlike previously described p38 inhibitors. The peptides only targeted TNF-mediated activation of p38. Consequently, it did not affect fMLF-stimulated respiratory burst in neutrophils and p38 activation. TNF binding to the TNF receptor was not affected by TNFRI_206–211,_ which also did not affect TNF-induced ERK1/ERK2, JNK and NF-κB activation, unlike TNF-neutralising biologics. These observations demonstrate that the TNFRI peptides are a unique class of agents that have the capability to block the actions of TNF that require p38, without inhibiting the p38 pathway in general, thus enabling the normal functioning of this pathway when it is activated by other stimuli. Intriguing is the finding that TNFR_206–211_ selectively blocks the ability of TNF to stimulate p38 activation, most likely explaining its unique in vivo effects in experimental inflammatory models.

Our work has illustrated that the TNFR peptide while capable of binding TNF does not prevent the binding of TNF to TNFRI but it selectively causes inhibition of p38 activation and associated function. Given that the TNFR peptide sequence covers the minimal region that is required for TNF to activate p38, it is likely the peptide alters the dynamics of the TNF–TNFR interaction—allowing not only TNF binding to TNFRI, but also the activation of the other signalling molecules that the receptor normally activates. This implies that other regions of TNFRI mediate the activation of the other signalling molecules. While this interaction is interesting, the mechanism leading to the selective inhibition  of p38 remains to be fully elucidated. Nevertheless, the data from the current study illuminates a novel approach to block the TNF-p38 signalling axis and treat inflammation. Another advantage of the TNFRI peptides over current anti-TNF biologics is the ability of the peptides to promote clearance of RSV from infected mice. It has been reported that dsRNA from RSV induces the production of inflammatory cytokines in bronchial epithelial cells via a p38 and not NF-κB-dependent mechanism^28^. It is therefore likely that the peptide by inhibiting the TNF-TNFRI-p38 axis prevents the development of a cytokine ‘storm’ and its wide range of debilitating effects on leukocyte function, enabling leukocytes to kill virus-infected cells effectively. In contrast, current anti-TNF biologics may interfere with virus-clearance and promote tissue damage. We have demonstrated that Ross River virus (RRV)-infected mice treated with etanercept showed decreased weight gain, higher viral titre in muscle, joints and blood, associated with a greater degree of tissue damage and inflammatory cell recruitment than untreated RRV-infected mice^29^. Thus, the TNFRI peptides appear to have a number of advantages over existing anti-TNF biologics and pharmacological p38 inhibitors. Although we cannot rule out the possibility that the TNF receptor biologics would not predispose the host to infections, it is less likely that this would be an issue because of their target specificity.

We propose that the TNFRI peptides could be developed as anti-TNF-p38 biologics for the treatment of inflammation. While peptide therapeutics are associated with disadvantages such as their susceptibility to proteolytic degradation in vivo and the need for injection, a number of peptides are currently undergoing or have completed phase 1, 2 or 3 clinical trials^30^. These include KAI-9803 (Kai Pharmaceuticals), a selective protein kinase C δ inhibitor peptide to reduce infarct size in subjects with ST elevation myocardial infarction; KAI-4169, a peptide agonist of the calcium sensing receptor for the treatment of secondary hyperparathyroidism; XG-102 (Xigen) (also known as AM-111 from Auris Medical), a JNK-inhibiting peptide composed of D-amino acids for the treatment of stroke, inflammation, severe inner ear disorders and acute sensorineural hearing loss; the islet neogenesis-associated protein (INGAP) peptide (Exsulin Corporation) for islet beta cell neogenesis and improvement in glycemic control in type 1 diabetic patients, and AZX100 (Capstone Therapeutics), a peptide derived from HSP20 for the regulation of smooth contraction and dermal scarring. Stability can be increased by the use of D-amino acids as shown by XG-102 (AM-111). Our in vitro data also show that D-TNFRI peptides exhibited increased activity over the L-forms (Fig. 3h versus Fig. 3j). Our finding also opens the possibility of developing nonpeptidyl small molecules to block the TNF-p38 axis. The feasibility of this approach is exemplified by SB 247464, a nonpeptidyl small molecule reported to activate the G-CSF receptor^31^.

In summary, we have identified an attractive approach to target key pathways in treating inflammation associated with microbial- or autoimmune-mediated immunopathogenesis. Selective targeting of discrete domains in TNFR and of key signalling pathways within the spectrum activated by TNFR has major advantages over the global inhibition of TNF–receptor interaction or the systemic inhibition of a specific signalling molecule and it is tempting to speculate that the concept derived from our findings will have a wider implication in other ligand–receptor interaction-based therapeutic intervention. Peptides developed in this manner could also serve as valuable tools for elucidating the mechanism of disease pathogenesis in vivo.

## Methods

### Mice

Female BALB/c mice, 6–8 weeks of age, were purchased from the Adelaide University Laboratory Animal Services, Adelaide, SA, Australia and from the Animal Resource Centre, Perth, WA, Australia. These were housed on a 12-h light/dark cycle with ad libitum access to food and water in the Women’s and Children’s Hospital Animal House, Adelaide, SA, Australia and the animal facility at the University of Canberra, ACT, Australia. The study was approved by the Women’s and Children’s Hospital Animal Ethics Committee, the University of Adelaide Animal Ethics Committee (project number AEC 782a/2/2011) and the University of Canberra Animal Ethics Committee (project number CEAE 08–13). The research was conducted according to the Animal Welfare Guidelines of the National Health and Medical Research Council of Australia.

### Neutrophils and cell lines

The study was approved by the CYWHS Human Ethics Committee (project number HREC 595/12/10). Neutrophils were prepared from peripheral blood of healthy donors, who had given informed consent, by a rapid single-step technique^32^. Blood was layered on to Hypaque-Ficoll (Pharmacia Biotech., Uppsala, Sweden), *d* = 1.114, and centrifuged at 600 × *g* for 20–30 min. After centrifugation, the leukocytes resolved into two bands and the neutrophils were harvested from the second band. The preparations of neutrophils were routinely of >99% viability and >98% purity. These were either used as cells in suspension or as adhered neutrophils by adding the cells to plasma-coated plates as previously described^33^. The 70Z/3 pre-B cell line, kindly provided by Dr. W. Langdon, University of Western Australia, Perth, Australia, are mouse pre-B lymphocytes which lack binding sites for human TNF and are non-responsive to the cytokine^18,19^. The cells were maintained in RPMI 1640 medium with 4.5 g/L glucose, 2 mM L-glutamine, 50 mM 2-mercaptoethanol and 10% FCS in an atmosphere of 95% air and 5% CO_2_. The monocytic cell line, MonoMac 6 was kindly provided by Dr. H.W.L. Ziegler-Heitbrock (Institute of Clinical Chemistry and Pathobiochemistry, Klinikum rechts der Isar, Technische Universitat, Munchen, Germany) and maintained in RPMI 1640 supplemented with 10% heat-inactivated foetal calf serum, 1 mM pyruvate, glutamine and antibiotics. A mouse fibroblastic fibrosarcoma cell line WEHI-164, clone 13 (from the ATCC) was obtained from Dr. Geeta Chauhdri (John Curtain School of Medical Research, Australian National University, Canberra, Australia). The cells were maintained in RPMI-1640 medium as above and the cells were detached with 0.25% trypsin EDTA. HEK 293T cells were maintained in DMEM, supplemented with 10% foetal calf serum. Cells, stably transfected with pRK5-TRAF2-FLAG, pRK5-TRAF2_87–501_-FLAG (Dr. V. Dixit, Genentech Inc., South San Francisco), or an empty plasmid (pRK5, BD Pharmingen/Biosciences, North Ryde, NSW, Australia) using LipofectAMINE 2000 (Life Technologies Pty Ltd, Mulgrave, VIC, Australia), were obtained from Dr. P. Xia, Centenary Institute of Cancer Medicine and Cell Biology, Sydney, Australia. The promyelotic leukaemia HL-60 cells (ATCC # CCL-240; batch # F-13704) were cultured in RPMI-1640 supplemented with antibiotics, L-glutamine and 10% heat-inactivated foetal calf serum. Twelve-well culture plates were seeded with 1–2 × 10^6^/ml HL-60s per well and the media supplemented with 1.25% v/v DMSO and allowed to differentiate for 4–5 days^34^, before treatment with TNF/peptides.

### TNF and TNF receptors

Human recombinant TNF was a gift from Dr. G.R. Adolf (Ernst-Boehinger Ingelheim Institut, Vienna, Austria) and also purchased from ProSpec-Tany TechnoGene (Rehovot, Israel). Recombinant mouse TNFα was purchased from Gibco (Frederick, MD). TNF stock solution was stored at −20 °C until required. Fresh dilutions of TNF were prepared in HBSS just prior to use. Human recombinant soluble TNFRI [rHusTNFRI (22–211), prepared as a chimeric protein with the 6x histidine tagged Fc portion part of human IgG_1_] was purchased from Prospec-Tany TechnoGene (Rehovot, Israel).

### TNF and TNFRI peptides

TNF_70–80_ and its control (GGDPGIVTH) were synthesised by Auspep Pty Ltd, (Tullamarine, VIC, Australia) and Genscript Biotech (Piscataway, NJ, USA). While it is difficult to directly equate the TNF activity to TNF_70–80_ because of the lack of tumour cell cytotoxicity of this peptide, comparisons on their ability to stimulate nitric oxide production in macrophage have been used. These show that 5.0 μg/ml of TNF_70–80_ equates to 1000 U/ml of TNF^9^. The sequence from the natural TNF-α was modified by substitution of isoleucine for leucine at residue 79. The modification extended the serum half-life of the peptide to >90 min and increased water solubility. Fresh dilutions of the peptide were prepared daily in HBSS or HBSS with 1% BSA. The His-tagged M4 (HM4) ∆HM4 (LKPGTT) was  purchased from Auspep Pty Ltd (Tullamarine, VIC, Australia). TNF_132–150_ (LSAEINRPDYLDFAESGQV), TNFRI_209–211_ peptide (GTT); scrambled control (TGT) and TNFRI_206–211_ (EDSGTT), scrambled control (GEDTST), TNF198–211 (GIENVKGTEDSGTT) D-forms, as well as peptides of sequences in CRD of TNFRI: TNFRI_77–82_ from CRD1 (QDTDCR), TNFRI_90–95_ from CRD2 (TASENH) and TNFRI_129–134_ from CRD3 (KNQYRH) were purchased from GenScript Biotech (Piscataway, NJ, USA). Peptides were purified by high-performance liquid chromatography (HPLC) and analysed by mass spectrometry to be >85% pure. Peptides were stored at −20 °C and solutions were made in HBSS or RPMI prior to use.

### Preparation of cell lysates

The cells were lysed in 200 μl of buffer A (20 mM HEPES, pH 7.4, 0.5% (v/v) Nonidet P-40, 100 mM NaCl, 1 mM EDTA, 2 mM Na_3_VO_4_, 2 mM dithiothreitol, 1 mM phenylmethylsulfonyl fluoride and 10 μg/ml leupeptin, aprotonin, pepstatin A and benzamidine) for 2 h at 4 °C with constant mixing^35^. After centrifugation (12,000 × *g* for 5 min), the supernatants were collected and the protein content of the lysates was determined by Lowry’s method of protein determination. Samples were stored at −70 °C until assayed. For western blot analysis, samples were mixed with Laemmli buffer and boiled before being stored for subsequent electrophoresis.

### Immunoprecipitation of signalling molecules

Lysates containing equal amounts of protein (0.5–1 mg) were pre-cleared with 15 μl/sample protein A sepharose (Pharmacia, NJ, USA) (1:1 slurry of sepharose in 20 mM Tris/HCL, pH 7.4) (4 °C) before being incubated with rabbit polyclonal anti-mouse p38α antibody (sc-535, Santa Cruz Biotech, Santa Cruz, CA) (3 μg/sample). After mixing for 2 h (4 °C), the immune complexes were precipitated by the addition of 20 μl/sample protein A sepharose. The immunoprecipitates were collected by centrifugation (16,000 × *g* for 15 s) and washed once with buffer A (4 °C), once with buffer B (10 mM Tris/HCl, pH 7.6, 100 mM NaCl, 1 mM EDTA and 100 μM Na_2_VO_4_) and once with assay buffer (20 mM Hepes, pH 7.2, 20 mM β-glycerophosphate, 3.8 mM *p-*nitrophenyl phosphate, 10 mM MgCl_2_, 1 mM dithiothreitol, 50 μM Na_3_VO_4_ and 20 μM ATP)^35^. The assay buffer was removed following a further centrifugation in preparation for kinase activity assay.

### Western blotting for MAP kinases and IκB-α

Western blot analysis was conducted as described previously^35^. Briefly, equal amounts of denatured protein from each lysate were separated by 10% SDS polyacrylamide gels. The proteins were electrophoretically transferred to nitrocellulose membranes using a Bio-Rad Trans-Blot Turbo (Hercules, CA, USA). The amounts of phosphorylated p38, ERK1/ERK2, JNK and levels of IκB-α were detected using rabbit polyclonal anti-p-p38 (Thr 180/Tyr 182)-R (sc-17852-R), mouse monoclonal anti-p-ERK1/2 (pT202/pY204.22A) (sc-136521), mouse monoclonal anti-p-JNK (G-7) (sc-6254) and rabbit polyclonal anti-IκBα (C-21)(sc-371) antibody (Santa Cruz Biotech, Dallas, TX, USA), respectively. These antibodies were used at 1:1000 dilution. Immune complexes were visualised by enhanced chemiluminescence. Ponceau S (0.1% in 5% acetic acid) staining was performed to verify total protein loading. Some blots were stripped and re-probed with rabbit polyclonal anti-p38 (C-20) (sc-535, Santa Cruz, used at 1:1000) antibody to confirm equal loading.

### Assays for p38 activity

p38 activity was determined, after immunoprecipitation, as described using myelin basic protein (MPB) as a substrate for the kinase in the presence of ^32^P-ATP^35^. We have demonstrated that p38 immunoprecipitates do not contain ERK1/ERK2^35^. Briefly, 30 μl of assay buffer (30 °C) containing 10 μCi of γ^32^P-ATP, 3.8 mM *p-*nitrophenyl phosphate and 15 μg of myelin basic protein was added to each sample. The samples were then incubated in a water bath at 30 °C. After 15 min, the assay was terminated by the addition of Laemmli buffer and boiling the samples (100 °C) for 5 min, Following fractionation (12% SDS polyacrylamide gels), the amount of ^32^P-MBP was determined using an Instant Imager (Packard Instruments, Canberra, Australia).

### Construction of TNFR1 mutants

The hTNFRI plasmid (pADB-TR55) was kindly provided by Dr. M. Kronke^19^ (University of Cologne, Koln, Germany). The signal sequence from the TNFR1 cDNA was isolated by PCR and cloned, via an NheI site incorporated into the 5′ primer and a KpnI site in the 3′ primer, into the expression vector pcDNA3.1 (ThermoFisher Scientific, Scoresby, VIC, Australia). A double-stranded oligonucleotide encoding His 6-tag, with KpnI and BamHI sticky ends, in frame with the signal peptide, was cloned downstream of the signal sequence. The transmembrane and cytoplasmic domains of the TNF receptor were also isolated by PCR and cloned, via a 3′ BstXI site and a 5′ XhoI site incorporated into the PCR primers, 3′ of the hexahistidine sequence. This construct was designated cDNA3sigHIScTNF (WT). The mutants designated M1 and M4 (Fig. 3) which lacked the first and all four cysteine-rich domains, respectively, were isolated using the restriction enzyme sites incorporated into the primers, cloned between the BamHI and BstXI sites of the pcDNA3sigHIScTNF, such that the reading was contiguous with the signal, 6x His and transmembrane/cytoplasmic coding sequences.

### Binding of TNF_70–80_ to recombinant human soluble TNFRI

Microtitre plates were coated with 100 μl/well of 3 μg/ml rHusTNFRI (ProSpec-Tany TechnoGene Ltd, Rehovot, Isreal) in 18MQ-cm H_2_O overnight at 4 °C and blocked with 1% BSA and 0.05% Tween-20 in PBS for 1 h at 4 °C. Aliquot of 10 μM of biotin-labelled TNF_70–80_ (Auspep Pty, Ltd., Tullamarine, VIC, Australia) plus various concentrations of unlabelled TNF_70–80_ or control peptide were added to each well and incubated for 3 h at 4 °C. The wells were washed four times with wash buffer (PBS containing 0.05% Tween-20) and the plates were incubated with 100 μl/well of poly-horseradish peroxidase streptavidin conjugate (1/6000 dilution in 1% BSA and 0.05% Tween-20 in PBS) for 45 min at 4 °C. Wells were washed four times with wash buffer and bound enzyme detected by the addition of 3′,3′,5′,5′-tetramethylbenzidine (TMB) substrate (Sigma-Aldrich, Castle Hill, NSW, Australia). Absorbance was measured using dual filter at 570/450 nm on a Dynatech MR700 Plate Reader (Guernsey, Channel Islands).

### TNF or TNF_70–80_ binding to TNFRI peptides

Depending on the experiment, microtitre plates were coated with 100 μl/well of either 3 μg/ml TNFRI_198–211_ in MilliQ water or TNFRI_206–211_ or the corresponding scrambled peptide in carbonate-bicarbonate buffer (pH 9.6) overnight at 4 °C and blocked with 1% BSA and 0.05% Tween-20 in PBS for 1 h at 4 °C. In the TNFRI_198–211_-coated plates, various concentrations of biotinylated TNF_70–80_ or control peptide were added to triplicate wells and incubated for 3 h at 4 °C. In the TNFRI_206–211_/scrambled peptide-coated plates, 50 U of TNF was added to triplicate wells, followed by washing with wash buffer (PBS/0.05% Tween-20) and then incubation with 50 μl of 1.5 μg/ml biotinylated monoclonal anti-TNF antibody (clone 2TNF-H33, ThermoScientific, Waltham, MA, USA). The wells from all plates were washed four times with wash buffer, then incubated with 100 μl/well of poly-horseradish peroxidase streptavidin conjugate (1/6000 dilution in 1% BSA and 0.05% Tween-20 in PBS) (ThermoScientific, Waltham, MA, USA) for 45 min at 4 °C. Wells were washed four times with wash buffer and bound enzyme detected by the addition of TMB substrate (Sigma-Aldrich, Castle Hill, NSW, Australia). Absorbance was measured using dual filter at 570/450 nm on a Dynatech MR700 Plate Reader (Guernsey, Channel Islands).

### TNF binding to soluble TNFRI in the presence of TNFRI_206–211_

TNF binding to sTNFRI was assayed by a modification of the Davis et al.^36^ method. Using a 96-well high-binding plate, 100 μl of 0.25 μg/ml sTNFRI in 0.05 M carbonate-bicarbonate buffer (pH 9.6) was incubated overnight at room temperature. The plate was washed three times with wash solution (PBS + 1% Tween-20) for 5 min each and then blocked for 1 h at 37 °C with 100 μl of PBS + 10% BSA. Following another three washes, the plate was loaded with 50 μl of either assay solution (PBS + 4% BSA), or 500 U TNF and/or 10 mM TNFRI_206–211_ (pre-incubated for 10 min together or separately) in assay solution and incubated for 2 h at room temperature. After three washes, all wells were loaded with 50 μl of 1.5 µg/ml biotinylated monoclonal anti-TNF antibody (Clone 2TNF-H33, ThermoScientific, Waltham, MA, USA) for 60 min, washed three times, then incubated with 50 μl of poly-HRP streptavidin conjugate (1/6000 in assay solution) for 30 min. After washing three times, 50 μl of TMB substrate was added and incubated for 15 min, followed by addition of 50 μl 0.5 M sulphuric acid. Absorbance was measured at 570/450 nm.

### Inhibition of TNF-induced p38 activation by TNFRI_206–211_

In these experiments, either 100 µl of 200 U/ml human TNF was treated with 100 µl of 4 mM of TNFRI_206–211_ or 500 µl of 100 ng/ml mouse TNF was treated with 500 µl of 8 mM of the TNFRI_206–211_ for 15 min at 37 °C, then added to human or mouse cells (2 × 10^6^) in a final volume of 1 ml or 5 ml, respectively. Following incubation at 37 °C for 15 min (for p38, ERK and IκB-α) or 30 min (for JNK), cell lysates were prepared for western blot analyses. Adherent neutrophils were required for ensuring ERK activation by TNF. Similarly, HL-60 cell-derived neutrophils were required for inducing JNK activation by TNF.

### Transfection of pre-B cells with hTNFRI or mutant receptor DNA

70Z/3 cells were plated onto 10 cm plates at 4 × 10^7^ cells in 15 ml culture medium. Before performing transient transfection, plasmid DNA (20 μg in 1 ml of RPMI 1640) was mixed with LipofectAMINE 2000 (Life Technologies Pty Ltd, Mulgrave, VIC, Australia) (60 μl in 1 ml of RPMI 1640) and the mixture was left at room temperature for 20 min. The mixture was directly added to the cells and incubated for 24 h. The cells were washed twice with HBSS before use.

### Cellular functions

Neutrophil superoxide production was measured by an indirect assay involving the reduction of the lucigenin, (9, 9′-bis *N*-methyl-acridinium nitrate) (Sigma-Aldrich, Castle Hill, NSW, Australia) and measuring the light output by lucigenin-dependant chemiluminescence^37^. Neutrophils (5 × 10^5^) were incubated with TNF_70–80_, TNF or other stimuli that had previously been pre-incubated in the absence or presence of receptor peptides for 20 min. After incubation, 500 μl of lucigenin (250 μM, final) was added and the final volume was adjusted to 1 ml. The cells were placed in luminometer (Autolumat Plus Model LB 953, Berthold Technologies, Bundoora, VIC, Australia) and the resulting chemiluminescence (in relative light units, RLU) measured. The results are expressed as maximal/peak rate of chemiluminescence produced unless specified otherwise. CR3 expression on neutrophils was measured by flow cytometry using anti-CD11b antibodies^33^. Neutrophils (1 × 10^6^) were incubated for 30 min with TNF that had previously been pre-incubated in the absence or presence of receptor peptides for 20 min. After treatment, 2 μl of PE-labelled mouse IgG_2a_ isotype control (Clone X39) or PE-labelled monoclonal anti-CD11b antibody (Clone D12) (both from BD Biosciences, North Ryde, NSW, Australia) was added to neutrophils in 200 μl of Isoton II (BD Biosciences) supplemented with 0.1% (w/v) BSA. After 30 min the cells were washed, fixed with formaldehyde (0.1%) and analysed on a FACScan (BD Biosciences, North Ryde, NSW, Australia). Cytokine (IL-1β and IL-8) mRNA productions were measured by quantitative RT-PCR. Briefly, total RNA was extracted from 1 × 10^6^ neutrophils using the TRIzol reagent (Life Technologies Australia Pty, Mulgrave, VIC, Australia) according to the manufacturer’s instructions. RNA was converted to cDNA using iScript cDNA synthesis kit (Bio-rad, Hercules, CA, USA). The cDNA was then amplified in triplicate reactions with iQ SYBR Green supermix (Bio-rad, Hercules, CA, USA) and 500 nM of each primer pair for IL-1β (Forward primer 5′–3′: TCA TTG CTC AAG TGT CTG AAG C; Reverse primer 5′–3′: TCC TGG AAG GAG CAC TTC AT) and IL-8 (Forward primer 5′–3′: TCT GTG TGA AGG TGC AGT TTT G; Reverse primer 5′–3′: AAT TTC TGT GTT GGC GCA GT) and the house keeping gene glyceraldehyde-3-phosphate dehydrogenase (GAPDH) using iQ5 system with v.3.1 software.

### In vivo inflammation models

Four models of in vivo inflammation reactions were used to assess the anti-inflammatory activity of the TNFRI peptides. Female, 6–8-week-old BALB/c mice were utilised in these models: (i) lipopolysaccharide-induced peritoneal inflammation^38^, (ii) carrageenan-induced paw inflammation, (iii) antigen-induced delayed hypersensitivity^38^, (iv) RSV-induced lung inflammation^39^. Inflammation was gauged by either one or a combination of the following: tissue changes, cellular infiltration and cytokine levels. Animals were allocated randomly to the various groups. The studies were sufficiently powered based on power calculation at α of 0.05 and β of 0.2.

### Lipopolysaccharide-induced peritoneal inflammation

Mice were injected intraperitoneally (i.p.) with 50 µg of LPS (Sigma-Aldrich, Castle Hill, NSW, Australia) or HBSS^40^. At 24 h, the animals were killed and cellular infiltrates in the peritoneal exudates examined in Giemsa-stained smears. The total leukocyte and neutrophil number in the peritoneal exudates was enumerated using a hemocytometer chamber and the neutrophils from a differential count of the stained smears.

### Delayed-type hypersensitivity

The delayed-type hypersensitivity (DTH) response was conducted by injecting mice with sheep red blood cells (SRBC) (100 µl of 10% haematocrit) (Applied Biological Products, Aldinga Beach, SA, Australia) subcutaneously in the back^38^. Six days later, mice were challenged in the right hind footpad with 25 μl of 40% suspension of SRBC. The DTH response was determined 24 h post challenge by measuring the footpad thickness of the unchallenged vs. SRBC-injected footpads with a caliper on a micrometre screw gauge. The inflammatory response was expressed as the difference in thickness (mm) between the unchallenged and antigen-challenged footpads.

### Carrageenan-induced paw inflammation

Carrageenan-induced paw inflammation was induced by inoculating mice with 1 ml/kg of a 1% solution of carrageenan type IV (w/v) into the right hind footpad^39^. The swelling/inflammation was assessed 24 h later by comparing the thickness between the unchallenged vs. carrageenan challenged footpads as described above.

### RSV-induced inflammatory mouse model

BALB/c mice were housed in microisolator cages with their own food and water supply and were treated intranasally (i.n.) with three doses of 100 mg/kg of TNFRI_206–211_ or the control scrambled peptide administered on day −1, 1 and 3. On day 0, mice were infected i.n. with 1 × 10^6^ plaque forming units of RSV A2. Prior to all intranasal treatments and infections mice were anaesthetised i.p. with Alfaxan. On day 5, mice were killed.

### Respiratory syncytial virus propagation

The RSV strain A2 was propagated in Vero E6 cells. An 80% confluent T150 flask of Vero E6 cells was infected with RSV at a multiplicity of infection of 0.5 in a total volume of 6 ml of DMEM. Cells were infected for 2 h at 37 °C and 5% CO_2_. After 2 h, 8 ml of DMEM with 5% foetal bovine serum was added and flasks incubated at 37 °C and 5% CO_2_ for ~2–3 days or until 25–50% syncytia formation was observed. At harvest time all but 2 ml of media was removed and cells were scraped from the flask, transferred to a 50 ml tube and incubated on ice for 10 min. Cells were sonicated for 5 s at 25 W, three times, with a 2 min incubation on ice between each sonication. Cell debris was removed by centrifugation at 350 × *g* for 7 min at 4 °C and the supernatant containing the virus stored at −80 °C. The Vero E6 cells used were VERO 1008 (Ver 76, clone E6) originally sourced from ATCC (ATCC CRL-1586). Cells were routinely tested for mycoplasma by using the LookOut Mycoplasma PCR detection kit (Sigma-Aldrich cat no. MP0035)

### Bronchoalveolar lavage and cytospin

After killing, the left lung was ligated at the left main branch of the trachea using surgical thread, and the right lung lavaged twice with 0.5 ml PBS. BAL fluid was centrifuged at room temperature at 350 × *g* for 5 min and the supernatant collected and stored at −80 °C. The cell pellets were lysed with BD PharmLyse Lysing Buffer, washed with PBS and resuspended in a small volume of PBS. Total BAL cells were counted using a hemocytometer. Cells were centrifuged onto glass slides (Menzel-Glaser, Germany) using a cytospin (Rotofix 32 Hettich, Germany), fixed in methanol and stained with modified Giemsa. Cell differential percentages were determined by light microscopy.

### Histological analysis

A lobe of the non-lavaged lung was fixed in 10% formalin, embedded in paraffin and 3 μm sections stained with hematoxylin & eosin (H&E). Slides were scored by an observer blinded to the treatment groups. The severity of inflammation was quantitated on a 0–3 scale with uninfected control mice defined as 0, and 3 being the maximum inflammation score^41^.

### Mouse cytokine analysis

IL-4 and IFN-γ cytokine concentration was determined from the first BAL wash by ELISA kits from BD Biosciences and R&D systems, respectively, according to the manufacturer’s instructions.

### Virus titres

RSV from a lobe of the non-lavaged lung was determined by immunostaining. The lung was homogenised in PBS (containing penicillin, streptomycin and neomycin) using the TissueLyser II (Qiagen, Chadstone Centre, VIC, Australia), centrifuged at 1700 × *g* and the supernatant collected. Twenty-four-well tissue culture plates were seeded with Vero E6 cells in DMEM containing 5% FBS incubated overnight at 37 °C and 5% CO_2_ to obtain 80–90% confluency. Monolayers were washed with HBSS and serial dilutions of the lung supernatants, in DMEM, were added to the cell monolayers in quadruplicate. Plates were incubated at 37 °C in 5% CO_2_ for 1 h. The wells were overlaid with 1 ml of 2% methylcellulose media (DMEM containing antibiotics, 2% FBS, 20% HBSS and 2% methylcellulose (Methocel®MC, Sigma-Aldrich, Castle Hill, NSW, Australia) and incubated at 37 °C and 5% CO_2_. After 6 days, the overlay was removed and monolayers fixed with 1 ml of ice-cold acetone:methanol (60:40) for 10 min. Plates were air dried at room temperature and blocked in 5% skim milk powder diluted in PBS for 30 min at room temperature. Primary RSV monoclonal anti-F antibody 131–2A was added to wells for 2 h, followed by goat anti-mouse IgG-alkaline phosphatase secondary antibody for 1 h. All antibodies were diluted 1:1000 in 5% block and incubated at 37 °C. After each antibody, plates were washed three times with PBS containing 0.5% Tween-20. The assay was developed using the Alkaline Phosphatase Substrate Kit II (Vector Laboratories, USA) as per manufacturer’s protocol.

### Statistics and analysis

Results are presented as mean ± standard error of the mean (SEM) unless stated otherwise. The F-test or Brown–Forsythe test was used to examine homogeneity or equality of variance. For parametric data, differences were analysed by either one or two-tailed *t*-test, one sample *t*-test or one-way ANOVA followed by Dunnett’s multiple comparison test. For non-parametric data, a Mann–Whitney analysis (one or two tailed) (comparison between two groups), a Fischer exact test (one tail) or, either a Friedman test or a Kruskal–Wallis analysis was performed, followed by Dunn’s multiple comparison test. Differences were considered statistically significant when *p* < 0.05. Statistical analyses were performed on GraphPad Prism 5 software.

### Data availability

The authors declare that the data supporting the findings of this study are available within the paper and from the authors on request.
